# Complete Genome Sequence of Alistipes indistinctus Strain 2BBH45, Isolated from the Feces of a Healthy Japanese Male

**DOI:** 10.1128/MRA.01284-20

**Published:** 2021-01-14

**Authors:** Yusuke Ogata, Mitsuo Sakamoto, Moriya Ohkuma, Masahira Hattori, Wataru Suda

**Affiliations:** a Laboratory for Microbiome Sciences, RIKEN Center for Integrative Medical Sciences, Tsurumi-ku, Yokohama, Japan; b Microbe Division/Japan Collection of Microorganisms, RIKEN BioResource Research Center, Tsukuba, Ibaraki, Japan; c PRIME, Japan Agency for Medical Research and Development (AMED), Tsukuba, Ibaraki, Japan; d Graduate School of Advanced Science and Engineering, Waseda University, Shinjuku-ku, Tokyo, Japan; University of Maryland School of Medicine

## Abstract

The genus *Alistipes* is one of the members of the human gut microbiota. Here, we report the complete genome sequence of Alistipes indistinctus strain 2BBH45, harboring plasmid p2BBH45.

## ANNOUNCEMENT

The genus *Alistipes* is a strictly anaerobic, Gram-negative-staining member of the human gut microbiota. Several species have been reported, such as A. finegoldii, A. putredinis (1), A. onderdonkii, A. shahii (2), A. indistinctus (3), A. inops (4), A. communis, and A. dispar (5). Here, we report the genome of *A. indistinctus* strain 2BBH45, isolated from the feces of a healthy Japanese male in his 30s.

Unless otherwise specified, the manufacturer’s instructions were used for all procedures and reagents, and default parameters were used for all software. The experiments were conducted in accordance with the RIKEN ethics committee (approval no. Tsukuba 27-1). A fecal sample (0.5 g) was suspended in 4.5 ml of prereduced phosphate-buffered saline (PBS). The diluted fecal sample was plated onto Brucella agar supplemented with 5% (vol/vol) horse blood for 2 to 4 days of incubation at 37°C in an environment containing an H_2_/CO_2_/N_2_ (1:1:8, by volume) gas mixture. By screening the bacterial colonies with partial 16S rRNA gene sequencing using the BigDye Terminator v3.1 cycle sequencing kit with the Applied Biosystems SeqStudio Genetic Analyzer (Thermo Fisher Scientific) as described previously (6), Alistipes indistinctus strain 2BBH45 was identified and isolated after plating three times. The purified 2BBH45 strain was then grown in 500 ml of Gifu anaerobic medium (GAM) broth (Nissui) for 7 days at 37°C to prepare the genomic DNA. DNA extraction, based on enzymatic lysis, was performed as previously reported (7). DNA sequencing was performed using the Illumina MiSeq (2 × 300-bp paired-end format) and PacBio Sequel platforms, and the library was prepared using the TruSeq DNA PCR-free kit (target length, 550 bp) and the SMRTbell template prep kit v2.0 (target length, 10 to 15 kbp) without DNA shearing. The MiSeq reads were trimmed and filtered with a quality value (QV) of >20 using the FASTX-Toolkit v0.0.13 (http://hannonlab.cshl.edu/fastx_toolkit), and the Sequel reads were corrected using Canu v1.8 (8) with additional options (corOutCoverage=10000, corMinCoverage=0, corMhapSensitivity=high). *De novo* hybrid assembly of both sets of quality-checked reads was performed using Unicycler v0.4.8 (9), which contains a check of generated contigs for overlapping and circularization. Gene prediction and genome annotation of the generated contigs was performed using DFAST v1.2.4 (https://dfast.nig.ac.jp). A similarity search of the predicted proteins against the database of Clusters of Orthologous Groups (COG) of proteins was performed using BLASTp v2.9.0+ (10).

We obtained a total of 532,093,998 bases from 892,246 filter-passed MiSeq paired-end reads with an average length of 299.5 bp and a total of 98,975,791 bases from 8,296 corrected PacBio reads with an *N*_50_ value of 18,756 bp. The hybrid assembly of quality-filtered trimmed reads generated two circular contigs, corresponding to the *A. indistinctus* strain 2BBH45 chromosome and plasmid p2BBH45.

The *A. indistinctus* strain 2BBH45 chromosome is 3,012,737 bp long with 54.8% GC content and encodes 2,400 protein-coding genes, 43 tRNA genes, and 2 5S rRNA, 2 16S rRNA, and 2 23S rRNA genes. The 16S rRNA sequence had the highest similarity to that of *A. indistinctus* strain YIT 12060^T^ (= JCM 16068^T^) with a 100% identity match in the NCBI rRNA/ITS database.

Plasmid p2BBH45 is 82,848 bp long with 47.7% GC content, contains 61 protein-coding genes and no tRNA or rRNA genes, and shows no significant similarity (identity, ≥90%; coverage, ≥70%) with any genome in the NCBI nonredundant (nr) database. These data suggest that p2BBH45 is a novel plasmid.

### Data availability.

The complete genome sequence of Alistipes indistinctus strain 2BBH45 was deposited in DDBJ/ENA/GenBank under the accession no. AP023049.1, which is linked to the BioProject accession no. PRJDB9565, the BioSample accession no. SAMD00216626, and the DDBJ Sequence Read Archive (DRA) accession no. DRX208623 and DRX208624.
